# Pioneering Hand Hygiene: Ignaz Semmelweis and the Fight Against Puerperal Fever

**DOI:** 10.7759/cureus.71689

**Published:** 2024-10-17

**Authors:** Sheuli Paul, Shradha Salunkhe, Kasireddy Sravanthi, Shailaja V Mane

**Affiliations:** 1 Paediatrics, Dr. D .Y. Patil Medical College, Hospital and Research Centre, Dr. D. Y. Patil Vidyapeeth (Deemed to be University), Pune, IND

**Keywords:** antiseptic procedures, chlorinated lime, germ theory, hand hygiene, historical vignette, ignaz semmelweis, infection control, maternal mortality, medical innovation, puerperal fever

## Abstract

Hungarian physician Ignaz Semmelweis (1818-1865) revolutionized medical practice through his introduction of antiseptic procedures. This breakthrough disrupted the chain of infection among new mothers and their infants, leading to dramatically reduced mortality rates from puerperal fever across continental Europe. Semmelweis faced significant resistance and disbelief when he argued through meticulous, empirically-based evidence that proper hand hygiene may prevent infection. Semmelweis' pioneering work on infection prevention remains highly relevant even today, as evidenced by contemporary practices aimed at controlling the spread of disease and improving patient safety through improved hygiene. In this article, we look into the key moments in Semmelweis's life that led to his revolutionary discoveries as well as oppositions against them and his lasting impact on modern medicine.

## Introduction and background

Ignaz Semmelweis, a Hungarian physician, is often heralded as the "savior of mothers"[1]. His pioneering work in antiseptic procedures significantly reduced the mortality rates from puerperal fever, a deadly infection that plagued maternity wards in the 19th century. While his pursuit of hand hygiene initially met fierce opposition and disbelief among medical professionals, the practice eventually became a cornerstone of contemporary infection control practices.

**Figure 1:**
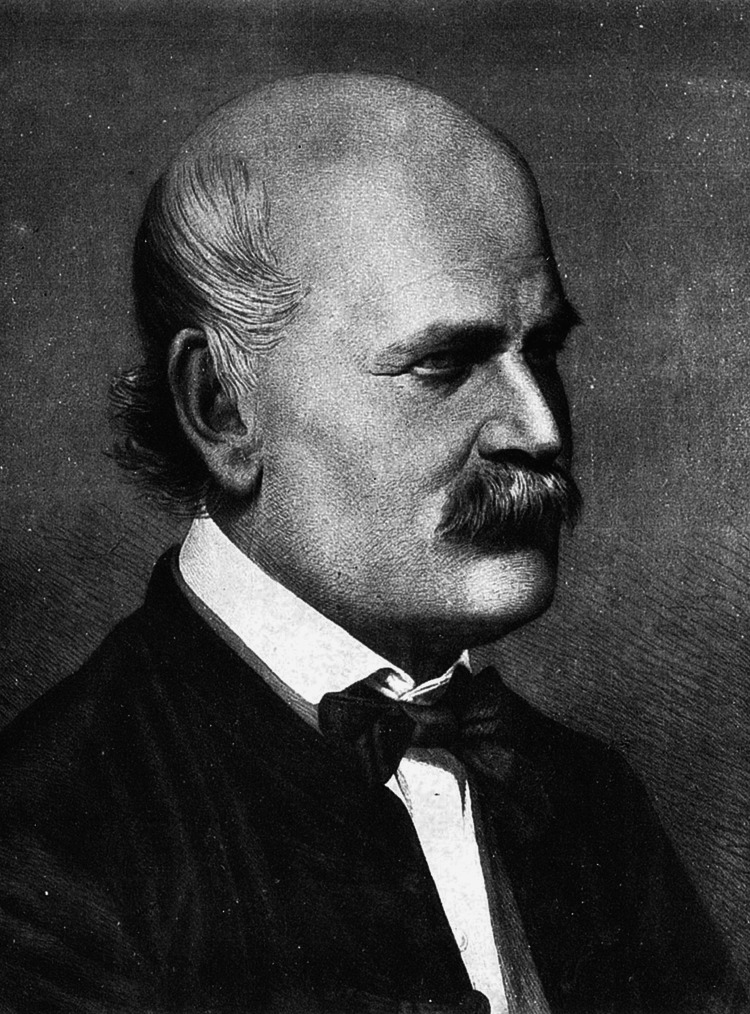
Ignaz Semmelweis (1860) aged 42 years Unknown author/wikimedia commons: public domain

Semmelweiss' ideas found few takers in the scientific establishments of his time, which led to a lifetime of professional ostracism. Interventions advocated by Semmelweis were only accepted after his death, as the germ theory of disease and antiseptic practices became widely recognized and better understood (Figure 1) [2]. Today he is widely hailed as a pioneer in the history of medicine for his extraordinary success in combating deadly diseases and saving countless lives simply by requiring doctors and healthcare workers to wash their hands.

## Review

Early life and education

He was born on July 1, 1818, in Buda, (now part of Budapest) Hungary. He was the fifth child of a prosperous family. Semmelweis first studied at the Law University, then changed to Medical School in order of priority for studies. He graduated in 1844 with a medical degree from the University of Vienna [3]. Semmelweis gained his interest in obstetrics and gynecology during his course at the University of Vienna. His early clinical mentors were among the leading medical minds of his day, and this shaped him to be significantly analytic in approaching a problem. He continued his studies in Vienna and gained excellent clinical experience - not least of which was in the obstetrics department.

Puerperal Fever

Throughout history, puerperal fever or child-bed fever, a dreaded outcome of delivery or abortion, has often resulted from infections in the genital tract that can progress to peritonitis, septicemia, pyemia, and death. Signs and symptoms are fever, abdominal pain, and septicemia. Despite the long-standing fear of the disease by past generations, mortality from puerperal fever was kept relatively low until lying in hospitals was created and operative obstetrics was developed. These supposed improvements led to regular internal exams for women in labor, unsterilized tools, bandages, fabrics, and overcrowding of patients. The lack of knowledge about asepsis led to the spread of infection and high mortality rates, and it was considered to be safer to deliver at home than in a hospital [4]. Outbreaks of childbed fever were documented, with the earliest well-known report dating back to 1646 regarding an outbreak at the Hoˆtel Dieu in Paris [5]. Reports of a similar nature were also received from the Allgemeines Krankenhaus in Vienna, which was home to Europe's biggest obstetric department at the time, and the Maternite´in Paris. Although the causes of puerperal fever were not well understood, knowledge of bacteria was nonetheless spreading, which would soon lead Joseph Lister to develop antisepsis as a means of avoiding puerperal fever (Lister instituted antiseptic techniques in 1867) [6].

The Scope of the Problem

The mortality rate of puerperal fever varied but was as high as 30% in some hospitals. Women who contracted the fever after childbirth typically experienced a rapid decline in health, leading to death within days. There was no knowledge of how it spread; hence, doctors could not stop it from spreading. This very typical and widespread problem was what motivated the medical world to try and figure out how they could cure it, which many tried but failed at due to the classical quantum forces of modern medicine [7].

Semmelweis's breakthrough

Semmelweis was transferred in 1846 to become an assistant at the First Obstetrical Clinic of Vienna General Hospital. During the period from 1840 to 1846, the first clinic exhibited a maternal mortality rate of 98.4 per 1,000 births, whereas the second clinic, staffed by midwives, demonstrated a substantially lower rate of 36.2 per 1,000 births (Table 1). This disparity was likely due to the greater incidence of induced or accelerated labor among women attended by medical students, who often went directly from the dissecting room to the delivery room. The significant difference in mortality rates between the two clinics perplexed Semmelweis and prompted him to investigate further [8].

**Table 1:** Puerperal fever, yearly mortality rates of the First and the Second Clinic at the Vienna General Hospital, 1841-1846 Source: Etiology, concept and prophylaxis of childbed fever (German: Die Ätiologie, der Begriff und die Prophylaxis des Kindbettfiebers) [9].

Year	First Clinic			Second Clinic		
	Births	Deaths	Rate	Births	Deaths	Rate
1841	3036	237	7.7	2442	86	3.5
1842	3287	518	15.8	2659	202	7.5
1843	3060	274	8.9	2739	164	5.9
1844	3157	260	8.2	2956	68	2.3
1845	3492	241	6.8	3241	66	2.03
1846	4010	459	11.4	3754	105	2.7
Total	20042	1989		17791	691	
Average			9.92			3.38

The Discovery

Based on his observations, Semmelweis realized that the medical students from the First Clinic went directly from conducting autopsies to the examination of pregnant women without washing their hands. One of the key factors that led Semmelweis to his groundbreaking discovery was the tragic death of his close friend. Namely, in 1847, Jakob Kolletschka, an autopsy professor with close relationships with Semmelweis, accidentally cut himself during the autopsy and soon died from sepsis. Although Semmelweis realized that the pathogen that caused sepsis was transferred from the student’s hands, he did not understand what infection he was dealing with and dubbed it by the term “cadaverous particles” [10]. To check his hypothesis, of which doctors ironically said that women would be safe if only men did not touch them, Semmelweis ordered all the medical personnel at the First Clinic to wash their hands with the chlorinated lime solution. The difference in mortality rates was enormous. For example, in 1847, the mortality rate of the First Clinic was 18.27%, whereas, in 1848, it had precipitously dropped to 1.27% [11].

The Handwashing Protocol and Its Advantages

Semmelweis's handwashing regimen, which involved thoroughly scrubbing hands with a chlorinated lime solution, was more effective than basic soap and water at removing organic matter and potential pathogens. This simple yet groundbreaking intervention significantly reduced the spread of infection, demonstrating that cleanliness could prevent the transmission of puerperal fever. Semmelweis's protocol was a significant improvement over previous practices that largely ignored hygiene in the clinical setting [12].

Detailed Analysis of the Results

Semmelweis's observations and subsequent implementation of handwashing with chlorinated lime significantly altered the course of medical practice in his clinic. The sharp decline in mortality rates provided robust evidence that cleanliness could prevent the transmission of infections. This experiment also highlighted the importance of empirical evidence in medical practice, challenging many of the existing beliefs held by his contemporaries [13]. In 1855, he relinquished his prior position to accept a professorship at the University of Pest. Six years later, in 1861, he published a scholarly monograph titled "The Etiology, Concept, and Prophylaxis of Childbed Fever" [9]. Yet, this groundbreaking work was unfortunately received with indifference by the medical establishment, who found the writing style inaccessible [13].

Resistance and opposition

Semmelweis faced significant resistance from his colleagues. The concept that they themselves might be the source of infection, and therefore emblematic of some degree of failure to maintain good standards both with regard to diligence in scrubbing, etc, alienated many of his colleagues. Unfortunately, Semmelweis failed to initially publish his findings in a comprehensive and compelling manner that would have convinced his medical peers. Instead, he relied heavily on providing direct instructions and implementing hospital-based protocols, which ultimately restricted the widespread adoption of his revolutionary discoveries. The medical community did not universally accept the germ theory of disease at that time, so it wasn't easy to explain Semmelweis's findings. Prominent physicians, such as Rudolf Virchow and Friedrich Scanzoni, despised Semmelweis. They did not want to believe that such destruction was caused by invisible particles on the hands of physicians. The resistance was also partly political; for instance, Semmelweis was a Hungarian physician in Vienna at a time when nationalist tensions were high. Such a thing as doctors washing their hands before each patient was a cumbersome tradition, and many could not accept the fact that their practices could harm their patients [4]. The broader medical community's reluctance to adopt Semmelweis’ practices had tragic consequences. Maternal mortality rates remained high in many hospitals, and countless women continued to die unnecessarily. Semmelweis himself grew increasingly frustrated with the rejection of his ideas.

Professional Consequences

His abrasive demeanor did not help ingratiate him with the medical authorities. He also used to give harsh statements of persons who were not in his favor, which further distanced him from other practitioners. This opposition ultimately led to his dismissal from the Vienna General Hospital. Undeterred, he tried to continue his scientific work in Hungary, but faced continued resistance from the authorities, which took a toll on his mental well-being [14].

Legacy and Impact

While Semmelweis died before his ideas were widely recognized, his work laid the foundation for future antiseptic progressions. Although many discounted the importance of hand hygiene for preventing infection, subsequent visionaries such as Louis Pasteur [15] and Joseph Lister recognized it and built on Semmelweis's work to elaborate a more complete set of antiseptic techniques.

Influence on Louis Pasteur and Joseph Lister

Semmelweis' established principles were the foundation upon which Louis Pasteur based his pioneering work in microbiology and which Joseph Lister utilized to develop antiseptic surgical techniques. Pasteur's discovery of germs as causing infection delivered the theory that had been absent in Semmelweis' attempt [16]. Lister also applied these principles to surgical practice and taught that carbolic acid was an antiseptic, used both with instruments post-operatively and wounds intra-operatively (before suturing), cutting back significantly on post-operative wound infection [17].

Modern Recognition

Semmelweis and his methods of antiseptic practices are sometimes presented as a few names that changed healthcare. International awards, institutions, and statues commemorate his contributions to medicine. Named after him is the Semmelweis University, in Budapest, which is one of Hungary’s leading medical schools. In 2015, the World Health Organization (WHO) declared May 5th as World Hand Hygiene Day, recognizing Semmelweis's role in promoting hand hygiene [18].

Continuing Relevance

Hand hygiene remains a critical concept in modern infection prevention practices, so revisiting the pioneering work of Semmelweis may be timely. The concepts that he first promoted form the basis for contemporary infection surveillance systems in human healthcare institutions globally. Hand hygiene is a cornerstone in infection and control strategies and was a very significant prevention method during the COVID-19 pandemic.

Statistical Evidence

Semmelweis obsessively monitored the death rate before and after he introduced his handwashing directive. He argued that the very low mortality rates from puerperal fever demonstrated by his data were very much in contrast to what was being seen, he presented these findings at several meetings. This hard evidence was among the most powerful elements of his case, but easily buried beneath theoretical squabbles that were also a feature of the time [19].

Broader Implications

The implications of Semmelweis's findings were not confined to obstetrics. They proposed a general principle that would be useful across different healthcare domains. The concept that doctors might serve as unwitting vectors for the mixing and seeding of infections was game-changing, ushering in a rethinking of how medicine is carried out across professions. This ripple effect is also clearly manifested in subsequent general healthcare and surgical antiseptic technique adoption.

Personal struggles and mental health

Decline and Institutionalization

In 1850, Semmelweis returned to Budapest, where he took up a position at St. Rochus Hospital. There, he continued to implement his handwashing protocols, achieving similarly impressive results, with maternal mortality dropping to less than 1%. He was eventually appointed as Professor of Theoretical and Practical Midwifery at the University of Pest in 1855. Despite these successes, Semmelweis struggled to gain broader acceptance for his methods. The rejection and criticism Semmelweis faced took a severe toll on his mental health. By the late 1850s, his behavior had become increasingly erratic, and he showed signs of severe depression and possible neurological disorder. In 1865, he was committed to an asylum, where he died under tragic circumstances shortly thereafter [20].

Posthumous Recognition

It actually took until after he had died for Semmelweis to get the credit that was his rightly due. Pasteur's adoption of the germ theory and Listerian antiseptic methods lent legitimacy to Semmelweis' views. Matters changed over subsequent years, however: historians and medical practitioners took a fresh look at Semmelweis’ legacy, which made clear that the scientist had played an important role in setting medicine on its modern course.

Semmelweis's legacy in modern medicine

Hand Hygiene Campaigns

Modern hand hygiene campaigns owe much to Semmelweis's pioneering efforts. This has led hospitals and healthcare facilities all over the globe to establish rigid handwashing protocols today as an effective infection containment measure [21]. The training programs for health care professionals incorporate the principles Semmelweis advocated, which recognize hand hygiene as a key safety measure of patient protection.

Educational Institutions and Awards

There are many medical institutions and one university that carry his name, helping to remember the achievements of Semmelweis. The fact that we cannot see beyond Semmelweis was well-validated by Richard Feynman when he concluded with the term 'Semmelweis Reflex', which describes our inability to accept what diminishes one's value towards noble tradition [22].

Ongoing Research

Current research in infection control and hospital hygiene continues to build on Semmelweis's work [23]. The investigations surrounding hand washing, the efficacy of different antiseptic agents, and hygiene practices on infection rates are the legacies that originated from Semmelweis's earlier studies. As a result, his legacy has now become part of the efforts to achieve better patient outcomes by improving hygiene.

## Conclusions

One of the first doctors to develop antiseptic procedures, Ignaz Semmelweis changed medicine and saved countless lives. He met barriers and the skepticism of contemporaries during his own lifetime, but he went on to make a huge impact in infection control. The legacy of Semmelweis serves as a powerful lesson to how hand hygiene should be considered in national and local policies while flagging up the critical need for multi-tiered infection control measures within healthcare settings. Ignaz Semmelweis' life is a living example of the value of scientific observation and the perseverance of research even in the face of opposition. The sphere of medicine and our comprehension of the prevention as well as cure against infections wouldn’t have been what it is today without him. Even as infectious diseases wax and wane, Semmelweis's principles are more pertinent than ever.
